# Conducting a health technology assessment in the West Bank, occupied Palestinian territory: lessons from a feasibility project

**DOI:** 10.1017/S0266462324000084

**Published:** 2024-01-15

**Authors:** Mervett Isbeih, Lieke-Fleur Heupink, Sharif Qaddomi, Rand Salman, Lumbwe Chola

**Affiliations:** 1 Palestinian National Institute of Public Health, Ramallah, Palestine; 2 Global Health Cluster, Division for Health Services, Norwegian Institute of Public Health, Oslo, Norway; 3 Department of Health Management and Health Economics, University of Oslo, Norway, Oslo

**Keywords:** health technology assessment (HTA), West Bank occupied Palestinian territory, virtual HTA, adaptive HTA, LMIC

## Abstract

**Objectives:**

To achieve universal health coverage (UHC), countries must make difficult choices to optimize the use of scarce resources. There is a growing interest in using evidence-based priority setting processes, such as Health Technology Assessment (HTA), to inform these decisions. In 2020, the Palestinian Institute of Public Health (PNIPH) and the Norwegian Institute of Public Health (NIPH) initiated a pilot to test the feasibility of coproducing an HTA on breast cancer screening in the West Bank, occupied Palestinian Territory. Additionally, a secondary aim was to test whether using an adaptive HTA (aHTA) approach that searched and transferred published evidence syntheses could increase the speed of HTA production.

**Methods:**

The applied stepwise approach to the HTA is described in detail and can be summarized as defining a core team, topic selection, and prioritization; undertaking the HTA including adaptation using tools from the European Network for HTA (EUnetHTA) and stakeholder engagement; and concluding with dissemination.

**Results:**

The aHTA approach was faster but not as quick as anticipated, which is attributed to (i) the lack of availability of local evidence for contextualizing findings and (ii) the necessity to build trust between the team and stakeholders. Some delays followed from the COVID-19 pandemic, which showed the importance of good risk anticipation and mitigation. Lastly, other important lessons included the ability of virtual collaborations, the value of capacity strengthening initiatives within low- and middle-income countries (LMICs), and the need for early stakeholder engagement. Overall, the pilot was successfully completed.

**Conclusion:**

This was the first HTA of its kind produced in Palestine, and despite the challenges, it shows that HTA analysis is feasible in this setting.

## Introduction

There is a growing demand for the institutionalization of evidence-informed priority setting (EIPS) in low- and middle-income countries (LMICs), to support the efficient and fair allocation of healthcare resources (1–3). EIPS is being championed as a means to achieving universal health coverage (UHC), because it requires the use of inclusive and transparent deliberative processes for supporting decision making (4;5). In many LMICs, healthcare resources and services are often not adequately available or accessible to a significant proportion of the population, due to supply and demand constraints, such as limited infrastructure, insufficient health personnel, and poor health-seeking behavior (6). In addition, the financial burden of accessing care can be catastrophic for many households (7). When allocation decisions are not evidence-based, they may result in an unfair distribution of resources, which can potentially impede progress in health care (8). Achieving UHC with well-designed efficient and equitable benefit packages can increase access to health care and consequently improve individual and population health. However, achieving UHC often remains a challenge for many countries financially, politically, and socially.

The sustainability of UHC requires the development and enforcement of various critical components, one of which includes the institutionalization of Health Technology Assessment (HTA). The 67th World Health Assembly held in 2014 recognized HTA as a relevant process and tool for supporting UHC, as it provides decision makers with locally relevant high-quality information to facilitate evidence-informed decision making (9). HTA is a multifactorial process that uses explicit methods to evaluate the effectiveness, safety, monetary implications, and ethical, social, organizational, and legal issues of health technologies, which can be anything from medicines, vaccines, or public health interventions. The information gathered is synthesized and determines the value of the specific health technology at points in its lifecycle (10).

In Palestine, the Ministry of Health (MOH) has previously proposed initiating steps for establishing a HTA process (11). Elements of HTA and evidence-based practices can also be found in the formulation of the Palestinian essential drug list (12). However, there is still a need to consider the institutionalization of evidence-informed processes, through structured processes such as HTA, which can provide support to decision making, as well as improve transparency and accountability.

In September 2020, during the COVID-19 pandemic, the Palestinian National Institute of Public Health (PNIPH) in collaboration with the Norwegian Institute of Public Health (NIPH) initiated a pilot project to establish the feasibility of conducting HTA analyses in Palestine. The objective was to coproduce an HTA relevant to local policy makers and decision makers and in the process document any challenges and lessons that could be used to guide future endeavors. More importantly, there are no existing frameworks for HTA in Palestine, making this experience unique and informative to policy makers and interested stakeholders. In this paper, we share our experiences from the pilot project and the lessons learned from coproducing a HTA in the West Bank, occupied Palestinian Territory. We describe the approach to coproduce the HTA focusing on the steps and methods used to produce the HTA report and conclude with a discussion on lessons learned from this collaboration.

## The aHTA approach/approach

**Figure 1.**
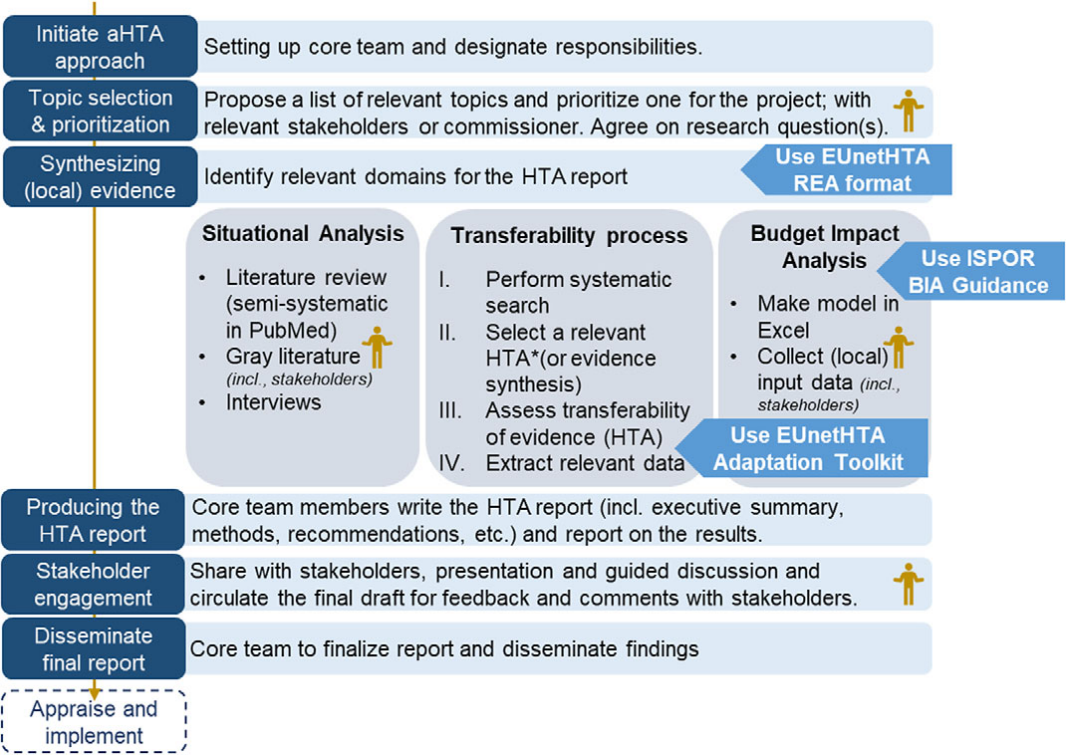
Steps taken to produce a locally relevant HTA. (see separate file) *Abbreviations: HTA = Health Technology Assessment, REA = Relative Effectiveness Assessment, EUnetHTA = European Network for HTA, ISPOR = Professional Society for Health Economics and Outcomes Research, BIA = budget impact analysis.

For the coproduction of this HTA, we set up a core team, selected a topic, wrote a protocol to guide the production of the HTA, and finally disseminated our findings. Below, we summarize the various steps taken (Figure 1) to adapt and produce a locally relevant HTA.

### Setting up the core team and designating responsibilities

To initiate the project, we first set up an appropriate team and work plan. The core team consisted of two experts from PNIPH with experience in evidence synthesis and two experts from NIPH with experience in HTA and health economics. Although many responsibilities were shared, PNIPH experts were primarily responsible for local data collection and mobilization of stakeholders within Palestine. Colleagues from NIPH were considered as the senior partners, initiating the main processes, executing the agreed-upon steps, and delivering the work plan. The core team met biweekly online to assess progress, divide tasks, and discuss the execution of plans. Throughout the project, attention was given to strengthening capacities using a learning-by-doing approach.

The core analysis team was carefully selected to complement the skills required for HTA, and we endeavored to develop and reinforce the core skills prior to the commencement of this work and during project execution. PNIPH and NIPH staff undertook a systematic review and meta-analysis course at NIPH in Norway. Additionally, a one-day introductory seminar on economic evaluation and budget impact analysis was given at NIPH to the core analysis team. The team also undertook an online short course on economic evaluation.

Essential materials and knowledge products were shared throughout the exercise, and informal lectures and workshops were given where appropriate. The biweekly meetings were also used as opportunities for sharing knowledge and clarifying any unclear concepts. In addition, the core group set up discussion forums on WhatsApp, allowing for quick responses to urgent questions. WhatsApp was an important application that allowed the team to communicate easily. Meetings took place on other online platforms including Zoom, Skype, and Microsoft Teams.

### Topic selection and prioritization

We selected a relevant topic by mapping disease priority areas for the MoH, mainly based on the burden of disease in early 2019, in a short evidence brief. Various highlighted issues included maternal and child health, cardiovascular diseases, and cancer (Supplementary File 1). Following further deliberation with PNIPH, it was decided to assess the appropriateness and potential impact of mammography screening, as this was considered an urgent priority by the MoH.

### Undertaking the HTA

The HTA production consisted of three components: (i) a situational analysis of the Palestinian context, including reviews of official reports and guidelines, stakeholder engagements, and interviews with patients and experts; (ii) a systematic search for evidence synthesis on the effectiveness of breast cancer screening; and (iii) a budget impact analysis showing the financial implications of breast cancer screening to the Palestinian government. To produce the HTA, we trialed the use of HTA adaptation instead of creating a *de novo* HTA. With HTA adaptation, we refer to the pragmatic use of HTA methodology, as in our case, where researchers reuse and transfer an existing HTA (or a relevant product underlying a HTA such as a systematic review or economic evaluation) produced in one context and adapted to inform another (local) context (13). The justification for using the adaptive HTA (aHTA) approach is that it is less time-consuming, requires fewer resources, and may thus be useful in contexts where expertise and resources are limited (14;15).

To identify relevant HTAs, and or underlying HTA products, a search strategy was developed by an information specialist at NIPH for the following databases: Epistemonikos, PubMed, and the Cochrane Database of Systematic Reviews (Supplementary File 3). Additionally, a separate search was undertaken for international guidelines in various electronic databases and Web sites. Through this process, the European (EU) Breast Cancer Guidelines (16;17) were identified as the best available evidence that could be adopted or adapted to the Palestinian context.

To further guide our HTA adaptation, we used guidance from the EU Network for HTA (EUnetHTA) Core Model for Rapid Effectiveness Assessments (REA) and EUnetHTA Adaptation Toolkit to inform the transfer of the evidence in the EU Breast Cancer Guidelines (18;19). Both tools follow the HTA Core Model^®^, a modular structure that describes and divides the relevant information into nine domains: current use of the technology, technical characteristics, safety, clinical effectiveness, cost and economic effectiveness, ethical analysis, organizational aspects, patient and social aspects, and legal aspects (20). The REA guidance contains four of the nine domains (current use, technical characteristics, safety, and clinical effectiveness) and provides questions to scope and identify relevant information for each domain. We selected the relevant questions and used these to collect information from published and gray literature, as well as from local stakeholders and experts (Supplementary File 2). The EUnetHTA Adaptation Toolkit (version 5) was used in a similar fashion. However, the toolkit consists of two parts where the first part contains six speeding sifting questions to aid selection of the to-be-adapted HTA or evidence synthesis. The second part of the toolkit consists of questions on the relevance, reliability, and transferability of selected HTA domains. The questions in the toolkit were used as checklists and brainteasers to identify the need for adjustments or any limitations when transferring evidence.

The detailed methods of this HTA are included in the protocol, which is available on the Web site of NIPH (21). The results are included in the main report, which is available upon request at PNIPH and partially provided in Supplementary File 4.

### Stakeholder engagement and dissemination

Stakeholder engagement was an important aspect of this project, given that this was the first time that the HTA methodology was documented in the Palestinian context. Local stakeholders were engaged to ensure a sense of ownership, as well as the ability to provide locally relevant information. Consultation was done throughout the project, starting with the development of the protocol and work plan. The draft reports were shared widely within the PNIPH, MoH, and with other stakeholders. We actively requested feedback to ensure that the HTA would be relevant for policy makers in Palestine.

We conducted a virtual stakeholder meeting to understand the context of breast cancer screening and the associated services in Palestine at the multisectoral level, involving policy makers, healthcare practitioners, and technicians within and without the MoH. Our discussion on the findings from the HTA analysis was well received. We also received useful feedback on the draft report to enhance the presentation of the overall findings.

We disseminated our results at the Annual HTAi Conference in 2021 and 2022, wrote a newsletter for HTAi, and blogged our experiences on the Web site of NIPH (21). The project protocol was also widely shared.

The final HTA was condensed into a four-page executive summary to communicate the main findings and recommendations. This summary provided a simplified account of the project and was widely disseminated to stakeholders. Future steps would include the appraisal of the evidence and implementation; however, this was outside the scope of the project, which focused on using methods underlying HTA analysis. Dissemination was done locally and internationally.

## Discussion and lessons learned

We successfully produced an HTA using aHTA methods, undertaking situational analysis, and consulting with stakeholders. Initiating the HTA and setting up the core team were made easier due to the long-standing collaboration between PNIPH and NIPH. Members from PNIPH included in the core team had previous research stays at NIPH, making it possible to develop a good relationship and mutual understanding of the nature of the work. We found that this strengthened relationship made it easier to carry the collaboration even when physical contact was limited by the COVID-19 pandemic.

Then, we took time to build and reinforce the relevant skills in systematic review and economic evaluation, which are necessary to enable critical appraisal of the quality of the evidence to be adapted. Adapting a poor quality or nonrelevant HTA would be a waste of time and potentially dangerous because it could lead to misleading recommendations. Throughout this project, meetings were used to reinforce core skills in systematic searches, budget impact analysis, qualitative interviews, and dissemination. The core team was involved in dissemination activities such as making presentations of preliminary results at international conferences. This effort taken to develop and reinforce core HTA skills was significant and should be considered if adaptation is used as a means to produce HTAs.

At the beginning of the project, we hypothesized that using aHTA would be easier than conducting a de novo HTA and that it would take much less time to execute. However, the length of time that it took to undertake the HTA adaptation was not as “quick” as envisioned. The initiation of this project began in September 2020 and was expected to be completed by December 2020. This feasibility project was the first known attempt to conduct an HTA in the West Bank, and the lack of frameworks on what information is of interest for local decision making added to the time required for editing and facilitation. To make up for this gap, we used guidance from EUnetHTA, which was useful, but also extensive and detailed, requiring lengthy discussions and reviews by members of the core team. The EUnetHTA guidance included many questions and not all were answered in the EU Breast Guidelines, so additional searches for information and interviews with stakeholders were necessary to ensure relevance for Palestine.

The HTA analysis was affected by the COVID-19 pandemic, which was rightfully prioritized throughout the project, mostly affecting work plans and deadlines. The restrictions on movement due to the COVID-19 pandemic delayed stakeholder engagement, due to strict working conditions nationwide and a lack of virtual communication tools in some cases. Additionally, face-to-face interviews with breast cancer patients during the COVID-19 pandemic could not be conducted. The COVID-19 pandemic was an extreme event, but competing interest will always be a problem within limited resource settings. Hence, we do see the benefit of adapting the evidence on breast cancer screening. Not having to produce a systematic review anew did ensure that under the circumstances, we took less time to summarize findings on clinical effectiveness and provided more time to do additional research with the aim of contextualizing the findings.

Although COVID-19 was a challenge to be worked around, other obstacles such as the lack of local good-quality data affected the HTA analysis. Palestine has data infrastructure such as electronic registries in the public health system at both the primary and secondary levels, but the data produced may not always be optimal. For instance, our situational analysis revealed that there are limited local data on the burden of breast cancer. Primary studies do exist, but they are quite outdated, include a small specific sample of women, and are not peer-reviewed. To circumvent the data issues, we were able to rely on expert opinion and data from similar settings where necessary. Although in this case we were able to adapt the available evidence, future work may require more extensive resources and production of locally relevant and accurate evidence.

Primary research from the local context was of interest to policy makers and stakeholders in this study, who rightfully wanted to understand whether findings from studies elsewhere were applicable or what the challenges in the local setting were. This is not solely a question related to the Palestinian setting but also addresses a broader question of uncertainties in HTA and the generalizability of results from randomized controlled studies conducted in high-income settings. In our case, we were able to build a rapport with local experts who were able to corroborate the evidence and tie it to the prevailing local circumstances. The effort that it takes to build this rapport should be considered when adapting HTAs in settings like ours. During the HTA process, PNIPH and NIPH worked as an external partner to the MoH and engaged with various stakeholders throughout this pilot. Effective stakeholder engagement not only worked as a form of transparency with multiple actors, but it also served to encourage open, healthy dialog among stakeholders in the private and public sectors and facilitated good information exchange. Overall, stakeholders from governmental and nongovernmental sectors made contributions with the aims of one day creating a national policy on breast cancer screening. This was a positive indicator for future work and collaborations.

Nevertheless, we should have included a broader group of stakeholders from the MoH from the outset, particularly during the topic selection for this HTA, but this was not done. Given different priorities and experiences with mammography screening in general among the different health sector categories, we would have probably framed a better-refined research question. Although our question was still interesting, it may not have been the right question for this context. During the project, we learned that there were only 14 mammography machines in the governmental healthcare sector in the West Bank. This would cover around 10 percent of eligible women, making an invitational mammography screening program unfeasible. Furthermore, during the stakeholder meeting, other interesting questions arose regarding the high incidence of young women with breast cancer and the cost related to the implementation of early diagnosis strategies, which could have also been included in the initial project proposal. Hence, engagement with stakeholders even prior to starting an HTA to provide insight into the topic selection and methods underlying HTA could have increased acceptance and usability, but could have added to the time necessary to finalize this project due to the need to include primary research.

## Conclusion

This was the first time a HTA analysis was undertaken in the West Bank. We are of the view that the use of an aHTA approach limited duplication efforts and facilitated a faster finalization of the HTA in comparison with standard HTA methods. Strong knowledge of methods among the core team, as well as early and ongoing engagement with stakeholders, was essential. Local evidence can provide insight into the applicability and transferability of recommendations and support buy-in from stakeholders, but data collection may increase time spent. The COVID-19 pandemic limited in-person contact, but simultaneously highlighted the ability of virtual collaborations. The collaboration provided an opportunity to strengthen the capacity for HTA and produce a HTA report in the West Bank, but more should be done to sustainably grow the capacity for undertaking HTA.

## Supporting information

Isbeih et al. supplementary material 1 Isbeih et al. supplementary material

Isbeih et al. supplementary material 2 Isbeih et al. supplementary material

Isbeih et al. supplementary material 3 Isbeih et al. supplementary material

Isbeih et al. supplementary material 4 Isbeih et al. supplementary material

## Supplementary material

The supplementary material for this article can be found at http://doi.org/10.1017/S0266462324000084.
