# Supplementary material for: Conducting a health technology assessment in the West Bank, occupied Palestinian territory: lessons from a feasibility project
Source: Int J Technol Assess Health Care. 2024 Jan 15;40(1):e12. doi: 10.1017/S0266462324000084 (PMC11569958; doi:10.1017/S0266462324000084)
Supplement: Isbeih et al. supplementary material 1 — Isbeih et al. supplementary material [file S0266462324000084sup001.docx]

# Supplementary file 1: Topic Selection

**Supporting capacity building for systematic reviews and evidence-based policy decision making in Palestine**

*E. Denison, E. Peacocke, Norwegian Institute of Public Health*

8 May 2019

Purpose

To describe possible topic areas for a systematic review that may be relevant for the Palestinian context. These possible topics have taken into consideration published literature and current information available related to burden of disease within the West Bank and Gaza Strip.

Objectives of the note

- Briefly summarise main thematic areas for a systematic review as identified in a targeted and rapid review of selected published literature.
- To be used as a first step in consultation with professionals at the Palestine National Institute of Public Health (PNIPH) and other relevant stakeholders to prepare for a consultation meeting for a systematic review topic (planned to be the week 24-27 June, 2019).
- To help the PNIPH reflect on the expertise of the stakeholders who should be invited to a consultation meeting for systematic review topic e.g. decision makers, clinical expertise relevant for the topics etc…

Background

The Norwegian Institute of Public Health (NIPH) is collaborating with PNIPH to support a systematic review to improve the evidence base for government officials and decision makers to set priorities at national level in Palestine. In November 2018, Rigmor Berg and Eva Denison lead two workshops on Systematic Reviews in Gaza and the West Bank. Following the workshops, the PNIPH indicated an interest in building their competence systematic reviews and would like to complete a review applicable for their local setting.

The approach of NIPH is to work collaboratively with PNIPH in completing a systematic review, which also includes facilitating the decision of possible topics that the review could include. The selection of a system review topic will be a multi-step process. The purpose of this document is to support the identification of a topic area, following this we identify the type of intervention, and level of the health system, primary, secondary or tertiary, etc… Then NIPH will search to see if there is a review previously published that is relevant and could be updated for the Palestinian setting.

**Burden of disease in Palestine**

NIPH has completed a targeted and rapid review of literature and we are suggesting the consideration of topics related to current research gaps and disease burden, as described below.

A recently published study from the region has noted that that there is a lack of association between primary research outputs and disease burden in Palestine (1). In terms of those topic areas with a high ratio of disease burden compared with a lower amount of research output in Palestine, a study found that maternal, neonatal and congenital conditions, cardiovascular disease, and cancer are topics where Palestine could benefit from further research (1). The same study (1) shows that the majority of research concerns questions of prevalence or association and that cross-sectional design dominate. Only a small proportion of research efforts concern preventative or therapeutic interventions evaluated in randomized controlled trials or controlled studies.

Years of life lost

Data from the Global Burden of Disease study 2016 (GBD; (2)) show that **neonatal pre-term birth complications**, **ischemic heart disease**, and **congenital anomalies** were the three leading causes of years of life lost (YLL) in Palestine in 2016.

According to the Palestinian Ministry of Health (3), cardiovascular disease was the main leading cause of deaths (30.6% of total deaths in 2016), followed by cancer (14.0%), stroke (12.8%), complications in the perinatal period (8.0%), and complications of diabetes (8.0%). The four causes of chronic non-communicable diseases including cardiovascular disease, cancer, stroke and diabetes accounted for 65.4% of all reported deaths in 2016.

Years lived with disability

**Maternal, neonatal and congenital conditions**, **cardiovascular disease**, and **musculoskeletal and neurological conditions** were reported as leading causes of years lived with disability (YLD) in Palestine in 2015 (2).

Data from the Global Burden of Disease study 2016 (4) show that migraine, major depressive disorder, and back pain were the three leading causes of YLDs in Palestine in 2016.

The apparent discrepancy between these two reports (2, 4) may be attributed to different data sets, or differences in calculating YLDs.

**Possible topic areas for a systematic review related to the Palestinian context**

- **Cancer**

In 2016, the most common cancer diagnoses in the West Bank were breast cancer (n. 388, 15.3% of total reported cancer cases), and colorectal cancer with 262 cases. Lung cancer was the most reported cancer case among males (13.6% of all reported cancer cases among Palestinian males in West Bank) (3).

- **Cardiovascular Disease**

Data concerning the West Bank in 2016 (4) show that 70% of all diseases of the circulatory system are cardiovascular diseases and 30% are cerebrovascular disease. Ischemic heart diseases (37.6%) and hypertensive disease (18.5%) are the most common cardiovascular diseases. Prevalence data for cardiovascular disease in Gaza was 9.7%, based on a sample of 2240 adults ≥ 25 years of age (5). Apparent discrepancies between West Bank and Gaza data may be attributed to different methods of sampling and data collection.

- **Neonatal pre-term birth complications, and maternal, neonatal and congenital conditions**

Maternal, neonatal and congenital conditions are the main cause of disability in Palestine (1). A published study found that the most common morbidity during pregnancy was related to haemorrhage, which can lead to organ failure (6). A recent study found that there was good coverage of the recommended clinical interventions in the study hospitals (for which Ramallah Hospital was one), which seems contrary to other findings that women are more likely to have a near miss^[[1]](#footnote-1)^ (7, 8) The quality of care in Palestine was noted as an area for further research (6).

**Examples of the type of focus for systematic review *N.B. examples for illustrative purposes***

- Treatment interventions (e.g. most effective interventions to treat postpartum haemorrhage)
- Clinical perception of an intervention (e.g. clinician compliance with standard treatment guidelines, or effects of guidelines related to rational use of medicines).
- Health system intervention (e.g. coverage of essential health interventions, or quality of service intervention).
- Other examples of interventions could be related to equipment, medicines or a diagnostic.

The examples given above can be thought of as “effect questions” where we typically would carry out a systematic review of effects of interventions at different levels in the health care system. They can also be thought of as topics where we would want to know “what is already done on this topic?” In that case, we would rather go for a systematic mapping review that uses the same rigorous methods and essentially the same process. The result will be a description of the research already carried out, in terms of methods and findings including identification of research gaps.

Based on the data reported above the NIPH encourages a discussion on and prioritization among the themes, with the aim to find a relevant topic for a systematic review. The NIPH will undertake scoping searches in the Cochrane Library and Epistemonikos database to identify published systematic reviews during the prioritization phase.

Next steps

- Skype call between PNIPH and NIPH to discuss concept note, workshop programme and answer any questions as soon as possible.
- PNIPH to consider the concept note and arrange consultation with relevant stakeholders to decide on the most suitable topic.
- PNIPH to identify members of the systematic review group.
- Skype call between PNIPH and NIPH (including members of the systematic review group) to prepare for the visit planned for week 24-27 June, 2019.

**References**

1. **Albarqouni L, Elessi K, Abu-Rmeileh Nmjhrp, Systems**. *A Comparison Between Health Research Output And Burden Of Disease In Arab Countries: Evidence From Palestine*. 2018;**16**(1):25.

2. **Naghavi M, Abajobir AA, Abbafati C, Abbas KM, Abd-Allah F, Abera SF, Et Al.** *Global, Regional, And National Age-Sex Specific Mortality For 264 Causes Of Death, 1980–2016: A Systematic Analysis For The Global Burden Of Disease Study 2016*. 2017;**390**(10100):1151-210.

3. **Palestine Ministry Of Health**. *Palestine Health Status Annual Report 2016* Nablus, Palestine: Ministry Of Health (Palestine), 2016.

4. **Vos T, Abajobir AA, Abate KH, Abbafati C, Abbas KM, Abd-Allah F, Et Al.** *Global, Regional, And National Incidence, Prevalence, And Years Lived With Disability For 328 Diseases And Injuries For 195 Countries, 1990–2016: A Systematic Analysis For The Global Burden Of Disease Study 2016*. 2017;**390**(10100):1211-59.

5. **Shahwan AJ, Abed Y, Desormais I, Magne J, Preux PM, Aboyans V, Et Al.** *Epidemiology Of Coronary Artery Disease And Stroke And Associated Risk Factors In Gaza Community–Palestine*. 2019;**14**(1):E0211131.

6. **Hassan SJ, Wick L, Dejong JJW, Birth**. *A Glance Into The Hidden Burden Of Maternal Morbidity And Patterns Of Management In A Palestinian Governmental Referral Hospital*. 2015;**28**(4):E148-E56.

7. **Bashour H, Saad-Haddad G, Dejong J, Ramadan MC, Hassan S, Breebaart M, Et Al.** *A Cross Sectional Study Of Maternal ‘Near-Miss’ Cases In Major Public Hospitals In Egypt, Lebanon, Palestine And Syria*. 2015;**15**(1):296.

8. **Say L, Souza JP, Pattinson Rcjbp, Obstetrics Rc, Gynaecology**. *Maternal Near Miss–Towards A Standard Tool For Monitoring Quality Of Maternal Health Care*. 2009;**23**(3):287-96.

1. Maternal Near Miss: is defined by the World Health Organization (WHO) as a woman who nearly died but survived a complication that occurred during pregnancy, childbirth or within 42 days of termination of pregnancy. [↑](#footnote-ref-1)
