# Supplementary material for: Conducting a health technology assessment in the West Bank, occupied Palestinian territory: lessons from a feasibility project
Source: Int J Technol Assess Health Care. 2024 Jan 15;40(1):e12. doi: 10.1017/S0266462324000084 (PMC11569958; doi:10.1017/S0266462324000084)
Supplement: Isbeih et al. supplementary material 2 — Isbeih et al. supplementary material [file S0266462324000084sup002.docx]

# Supplementary file 2: Questions to aid data extraction

Questions to guide extraction and adaptation. Adapted from EUnetHTA: HTA Core Model for Rapid Relative Effectiveness (1).

| **Topic** | **Question** |
| --- | --- |
| **Description and technical characteristics of the technology** | |
| Features of the technology | What types of mammography are available and are there other test/comparators? |
| Features of the technology | What is the claimed benefit of the mammography in relation to the comparator(s)? |
| Features of the technology | Who administers the mammography and the comparator(s) and in what context and level of care are they provided? |
| Investments and tools required to use the technology | What kind of special premises are needed to use mammography and the comparator(s)? |
| Investments and tools required to use the technology | What equipment and supplies are needed to use mammography and the comparator(s)? |
| Regulatory status | What is the reimbursement status of the mammography? |
| **Health problem and current use of technology** | |
| Target condition | What are the known risk factors for breast cancer? |
| Target condition | What is the natural course of breast cancer? |
| Target condition | What are the symptoms and the burden of disease or health condition for the patient? |
| Target condition | What are the consequences of breast cancer for the society? |
| Current management of the  condition | How is breast cancer currently diagnosed according to published guidelines and in practice? |
| Current management of the  condition | How is breast cancer currently managed according to published guidelines and in practice? |
| Target population | What is the target population in this assessment? |
| Target population | How many people belong to the target population? |
| Utilization | How much is mammography utilized? |
| **Safety** | |
| Patient safety | How safe is mammography in relation to the comparator(s)? |
| Patient safety | Are the harms related to frequency of mammography? |
| Patient safety | How does the frequency or severity of harms change over time or in different settings? |
| Patient safety | What are the susceptible patient groups that are more likely to be harmed through the use of mammography? |
| Patient safety | Are mammography and comparator(s) associated with user dependent harms? |
| Safety risk management | What kind of data/records and/or registry is needed to monitor the use of mammography and the comparator(s)? |
| Patient safety | What are the consequences of false-positive, false negative and incidental findings generated by using mammography from the viewpoint of patient safety? |
| **Clinical effectiveness** | |
| Mortality | What is the expected beneficial effect of mammography on mortality? |
| Morbidity | How does mammography affect symptoms and findings (severity, frequency) of breast cancer? |
| Morbidity | How does mammography affect progression (or recurrence) of the disease or health condition? |
| Function | What is the effect of mammography on patients’ body functions? |
| Function | How does the use of mammography affect activities of daily living? |
| Health related quality of life | What is the effect of mammography on generic health-related quality of life? |
| Health related quality of life | What is the effect of mammography on disease-specific quality of life? |
| Patient satisfaction | Were patients satisfied with mammography? |
| Test accuracy | What is the accuracy of mammography against reference standard? |
| Test accuracy | What is the optimal threshold value in this context? |
| **Costs** | |
|  | What is the price of the technology being assessed and its comparators? |
|  | What are the total costs per patient of the intervention in relation to its comparators? |
|  | What is the budget impact of the intervention? |
|  | What type of uncertainty is associated with cost and budget impact estimates? |
| **Cost-effectiveness** | |
|  | What are the expected benefits and costs associated with the technology being assessed and its comparators? |
|  | What is the incremental cost-effectiveness ratio (ICER) of the technology being assessed in relation to its comparators?  What type of uncertainty (methodological, structural and parameter) is associated with the ICER estimate? |
|  | Is the cost-effectiveness of the intervention different between patient groups (subgroups)? |
| **Other factors (when necessary)** | |
|  | Are there any ethical, organizational, social or legal aspects specific to the intervention that should be taken into consideration in assessment? |
|  | Is the intervention associated with any specific patient perspectives that should be taken into consideration in assessment? |

**Reference**

1. **EUnetHTA JAWP5**. *The HTA core model® for Rapid Relative Effectiveness Assessments (Version 4.2)*. 2015.
