# Supplementary material for: Conducting a health technology assessment in the West Bank, occupied Palestinian territory: lessons from a feasibility project
Source: Int J Technol Assess Health Care. 2024 Jan 15;40(1):e12. doi: 10.1017/S0266462324000084 (PMC11569958; doi:10.1017/S0266462324000084)
Supplement: Isbeih et al. supplementary material 3 — Isbeih et al. supplementary material [file S0266462324000084sup003.docx]

# Supplementary file 3: Search strategy

To produce the Health Technology Assessment (HTA) for Palestine, among other things a review of the international evidence on breast cancer screening was conducted. Below we describe the methods in more detail and provide the search strategy.

### Developing the search strategy

We adopted the methods of a similar study undertaken by the Norwegian Institute of Public Health (NIPH) to assess the impact of breast cancer screening in the Republic of Moldova (1). Inspired by the European Network for HTA (EUnetHTA) adaptation toolkit (2) to reduce duplication efforts, we used a strategy of identifying and adopting and existing evidence synthesis of high quality. A systematic search of the international literature was undertaken to identify systematic reviews and HTA of breast cancer screening. Other types of evidence syntheses including clinical guidelines, were considered if relevant and of high quality. Table 2 shows the inclusion criteria based on the Population, Intervention, Comparator, Outcome and study design (PICOS) format used to guide the literature search and inclusion of evidence.

Table 2: PICOS used to guide the literature search

| **Population:** | Asymptomatic women between the ages of 40 and 75 years |
| --- | --- |
| **Intervention:** | Mammography screening |
| **Comparison:** | No mammography screening |
| **Outcome:** | Morbidity, mortality, quality of life (from psychological effects and harms of screening, including: overdiagnosis (woman-perspective), rate of mastectomies, false positive related adverse effects measured as psychological distress, biopsies and surgeries. |
| **Study design:** | Recent, relevant, and high quality systematic review, or health technology assessment, or guidelines based on systematic reviews/evidence synthesis. |

The search strategy was developed by an Information Specialist (Librarian) at NIPH for the following databases: Epistemonikos, PubMed and the Cochrane Database of Systematic Reviews. An additional search was undertaken for international guidelines in various electronic databases and websites. The initial search was developed for the Moldova review and was undertaken between the 1st and 15th of September 2019 for systematic reviews and between the 15th and the 30th of January 2020 for international guidelines (1). These searches were updated in September 2020 for the Palestine review. Additional searches were conducted in the Intern The International Network of Agencies for HTA database in September 2020 to identify any relevant HTAs.

### Selection of evidence synthesis

The study selection and quality assessment using the A MeaSurement Tool to Assess systematic Reviews (AMSTAR) II checklist was used from the Moldavian project. Included full-text articles and guidelines were independently reviewed by two reviewers for final inclusion and data extraction. Any disagreements on eligibility between reviewers were resolved by consensus or the final judgement of a third reviewer. Relevant systematic reviews matching the research question were assessed for quality. Finally, the most recent high quality systematic reviews that corresponded to the PICOS and predefined outcomes was selected.

### Data extraction

The data extraction was guided by assessment elements table provided in the HTA Core Model for Relative Effectiveness Assessment (REA), version 3.0 (3). Data was extracted by one reviewer (LH) from the core team and checked by a second reviewer (LC). Any disagreements between reviewers were resolved through discussion. Further consultations on results extraction were made with experts at NIPH conducting the Moldova review. We adopted the quality assessment provided in the included studies.

### EUnetHTA Adaptation Toolkit

Both the speedy sifting and main toolkit from the EUnetHTA adaptation toolkit, version 5 (2), were used to assess the relevance, reliability, and transferability of the included reviews (speedy sifting) and extracted information (main toolkit). The questions in the main toolkit were mostly used as aids to ensure nothing was missed.

**References**

1. **Cumpana M, Buzdugan L, Anisei A, Otgon S, Gore M, Sofroni L, et al.** *Effect Of Breast Cancer Screening In The Republic Of Moldova [Unpublished Report]*. National Public Health Agency (Moldolva) and Norwegian Institute of Public Health, 2021.

2. **Guegan E, Milne R, Pordage A**. *EUnetHTA HTA Adaptation Toolkit & Glossary: Revised Version 5*. UK: EUnetHTA, 2011.

3. **EUnetHTA JAWP5**. *The HTA core model® For Rapid Relative Effectiveness Assessments (Version 4.2)*. 2015.

### Search Strategy

Database: PubMed

Date: 26.09.2019

Hits: 284

| Search | Query | Items found |
| --- | --- | --- |
| [#19](https://www.ncbi.nlm.nih.gov/pubmed/advanced) | Search (#16) OR #17 Filters: Publication date from 2016/01/01 to 2019/12/31 | [284](https://www.ncbi.nlm.nih.gov/pubmed/?cmd=HistorySearch&querykey=19) |
| [#18](https://www.ncbi.nlm.nih.gov/pubmed/advanced) | Search (#16) OR #17 | [866](https://www.ncbi.nlm.nih.gov/pubmed/?cmd=HistorySearch&querykey=18) |
| [#17](https://www.ncbi.nlm.nih.gov/pubmed/advanced) | Search (systematic[sb]) AND #14 | [334](https://www.ncbi.nlm.nih.gov/pubmed/?cmd=HistorySearch&querykey=17) |
| [#16](https://www.ncbi.nlm.nih.gov/pubmed/advanced) | Search (#14) AND #15 | [861](https://www.ncbi.nlm.nih.gov/pubmed/?cmd=HistorySearch&querykey=16) |
| [#15](https://www.ncbi.nlm.nih.gov/pubmed/advanced) | Search Meta-Analysis[Mesh:NoExp] or systematic* review*[Title/Abstract] or metaanal*[Title/Abstract] or meta anal*[Title/Abstract] or (review[Title/Abstract] and (structured search*[Title/Abstract] or database* search*[Title/Abstract] or systematic* search*[Title/Abstract])) or integrative review*[Title/Abstract] or evidence review*[Title/Abstract] | [314500](https://www.ncbi.nlm.nih.gov/pubmed/?cmd=HistorySearch&querykey=15) |
| [#14](https://www.ncbi.nlm.nih.gov/pubmed/advanced) | Search (#3) OR #13 | [54158](https://www.ncbi.nlm.nih.gov/pubmed/?cmd=HistorySearch&querykey=14) |
| [#13](https://www.ncbi.nlm.nih.gov/pubmed/advanced) | Search ((#6) OR #9) AND #12 | [23989](https://www.ncbi.nlm.nih.gov/pubmed/?cmd=HistorySearch&querykey=13) |
| [#12](https://www.ncbi.nlm.nih.gov/pubmed/advanced) | Search (#10) OR #11 | [381958](https://www.ncbi.nlm.nih.gov/pubmed/?cmd=HistorySearch&querykey=12) |
| [#11](https://www.ncbi.nlm.nih.gov/pubmed/advanced) | Search (breast cancer[Title/Abstract] OR breast neoplasm*[Title/Abstract] OR breast tumo*[Title/Abstract] OR mammary cancer*[Title/Abstract] OR malignant neoplasm* of breast[Title/Abstract] OR malignant tumo* of breast[Title/Abstract] OR breast malignant tumo*[Title/Abstract] OR cancer of breast[Title/Abstract] OR human mammary carcinoma[Title/Abstract] OR cancer breast[Title/Abstract] OR breast malignant neoplasm[Title/Abstract] OR breast malignant neoplasms[Title/Abstract] OR cancer mammary[Title/Abstract] OR cancers mammary[Title/Abstract] OR mammary Carcinoma[Title/Abstract] OR mammary Carcinomas[Title/Abstract] OR mammary neoplasm[Title/Abstract] OR mammary neoplasms[Title/Abstract] OR breast carcinoma[Title/Abstract] OR breast carcinomas[Title/Abstract] OR mammary tumo*[Title/Abstract]) | [313654](https://www.ncbi.nlm.nih.gov/pubmed/?cmd=HistorySearch&querykey=11) |
| [#10](https://www.ncbi.nlm.nih.gov/pubmed/advanced) | Search breast neoplasm[MeSH Terms] | [281275](https://www.ncbi.nlm.nih.gov/pubmed/?cmd=HistorySearch&querykey=10) |
| [#9](https://www.ncbi.nlm.nih.gov/pubmed/advanced) | Search (#7) OR #8 | [575858](https://www.ncbi.nlm.nih.gov/pubmed/?cmd=HistorySearch&querykey=9) |
| [#8](https://www.ncbi.nlm.nih.gov/pubmed/advanced) | Search (Ultrasound [Title/Abstract] OR Echography[Title/Abstract] OR Ultrasonic Imag*[Title/Abstract] OR Medical Sonography[Title/Abstract] OR Ultrasonic Diagnos*[Title/Abstract] OR Computer Echotomography[Title/Abstract] OR Ultrasonic Tomography[Title/Abstract] OR breast imaging*[Title/Abstract] OR ultrasonography[Title/Abstract] OR Sonography[Title/Abstract] OR sonography medical[Title/Abstract]) | [343687](https://www.ncbi.nlm.nih.gov/pubmed/?cmd=HistorySearch&querykey=8) |
| [#7](https://www.ncbi.nlm.nih.gov/pubmed/advanced) | Search ("Ultrasonography, mammary"[MeSH Terms]) OR "ultrasonography"[MeSH Terms] | [422458](https://www.ncbi.nlm.nih.gov/pubmed/?cmd=HistorySearch&querykey=7) |
| [#6](https://www.ncbi.nlm.nih.gov/pubmed/advanced) | Search (#4) OR #5 | [751901](https://www.ncbi.nlm.nih.gov/pubmed/?cmd=HistorySearch&querykey=6) |
| [#5](https://www.ncbi.nlm.nih.gov/pubmed/advanced) | Search magnetic resonance Imag*[Title/Abstract] OR magnetic resonance spectroscop*[Title/Abstract] OR magnetic resonance tomograph*[Title/Abstract] OR NMR[Title/Abstract] OR NMRs[Title/Abstract] OR MRI[Title/Abstract] OR MRIs[Title/Abstract] OR fMRI[Title/Abstract] OR fMRIs[Title/Abstract] OR MR tomograph*[Title/Abstract] OR MR imag*[Title/Abstract] OR MR scan[Title/Abstract] OR MR scans[Title/Abstract] OR Zeugmatograph*[Title/Abstract] OR chemical shift Imag*[Title/Abstract] OR proton spin Tomograph*[Title/Abstract] OR spin echo Imag*[Title/Abstract] OR diffusion Tractograph*[Title/Abstract] OR echo planar Imag*[Title/Abstract] OR echoplanar Imag*[Title/Abstract] OR magnetic resonance Angiograph*[Title/Abstract] OR magnetization transfer contrast Imag*[Title/Abstract] OR MR scanning*[Title/Abstract] OR magnetic resonance scan*[Title/Abstract] | [599173](https://www.ncbi.nlm.nih.gov/pubmed/?cmd=HistorySearch&querykey=5) |
| [#4](https://www.ncbi.nlm.nih.gov/pubmed/advanced) | Search ("Magnetic Resonance Imaging"[Mesh:NoExp] or "Diffusion Magnetic Resonance Imaging"[Mesh:NoExp] or "Diffusion Tensor Imaging"[Mesh:NoExp] or "Echo-Planar Imaging"[Mesh:NoExp] or "Fluorine-19 Magnetic Resonance Imaging"[Mesh:NoExp] or "Magnetic Resonance Angiography"[Mesh:NoExp] or "Magnetic Resonance Imaging, Cine"[Mesh:NoExp]) | [428952](https://www.ncbi.nlm.nih.gov/pubmed/?cmd=HistorySearch&querykey=4) |
| [#3](https://www.ncbi.nlm.nih.gov/pubmed/advanced) | Search (#1) OR #2 | [38287](https://www.ncbi.nlm.nih.gov/pubmed/?cmd=HistorySearch&querykey=3) |
| [#2](https://www.ncbi.nlm.nih.gov/pubmed/advanced) | Search (mammograph*[Title/Abstract] or xeromammograph*[Title/Abstract] or digital breast tomosynthes*[Title/Abstract]) | [28705](https://www.ncbi.nlm.nih.gov/pubmed/?cmd=HistorySearch&querykey=2) |
| [#1](https://www.ncbi.nlm.nih.gov/pubmed/advanced) | Search (mammography[MeSH Terms]) OR xeromammography[MeSH Terms] | [29082](https://www.ncbi.nlm.nih.gov/pubmed/?cmd=HistorySearch&querykey=1) |

Database: Cochrane Database of Systematic Reviews

Date: 26.09.2019

Hits: 61

| **Search** | **Query** | **Items found** |
| --- | --- | --- |
| #1 | [mh ^Mammography]] | 764 |
| #2 | [mh ^xeromammography] | 5 |
| #3 | (mammograph* or xeromammograph* or "digital breast tomosynthes*"):ti,ab | 1967 |
| #4 | #1 OR #2 OR #3 | 2095 |
| #5 | [mh ^"Magnetic Resonance Imaging"] | 6882 |
| #6 | [mh ^"Diffusion Magnetic Resonance Imaging"] | 237 |
| #7 | [mh ^"Diffusion Tensor Imaging"] | 119 |
| #8 | [mh ^"Echo-Planar Imaging"] | 82 |
| #9 | [mh ^"Fluorine-19 Magnetic Resonance Imaging"] | 0 |
| #10 | [mh ^"Magnetic Resonance Angiography"] | 434 |
| #11 | [mh ^"Magnetic Resonance Imaging, Cine"] | 229 |
| #12 | ("Magnetic Resonance Imag*" or "magnetic resonance spectroscop*" or "magnetic resonance tomograph*" or "NMR" or "NMRs" or "MRI" or "MRIs" or "fMRI" or "fMRIs" or "MR tomograph*" or "MR imag*" or "MR scan" or "MR scanning*" or "MR scans" or "Zeugmatograph*" or "Chemical Shift Imag*" or "Proton Spin Tomograph*" or "Magnetization Transfer Contrast Imag*" or "Spin Echo Imag*" or "Diffusion Tractograph*" or "Echo Planar Imag*" or "Echoplanar Imag*" or "Magnetic Resonance Angiograph*" or "Magnetic resonance scan*"):ti,ab | 22760 |
| #13 | #5 OR #6 OR #7 OR #8 OR #9 OR # 10 OR # 11 OR #12 | 967446 |
| #14 | [mh ^"Ultrasonography, Mammary"] | 72 |
| #15 | [mh ^Ultrasonography] | 4690 |
| #16 | ("ultrasound" or "echography" or "ultrasonic imag*" or "medical sonography" or "ultrasonic diagnos*" or "computer echotomography" or "ultrasonic tomography" or "breast imaging*" or "ultrasonography" or "Sonography" or "sonography medical"):ti,ab | 32158 |
| #17 | #14 OR #15 OR #16 | 33753 |
| #18 | [mh ^"breast neoplasm"] | 11727 |
| #19 | (“Breast cancer” or “breast neoplasm*” or “Breast Tumo*” or “Mammary Cancer*” or “Malignant Neoplasm* of Breast” or “Malignant Tumo* of Breast” or “Breast Malignant Tumo*” or “Cancer of Breast” or “Human Mammary Carcinoma” or ”mammary tumo*” or “cancer breast” or “breast malignant neoplasm*” or “cancer* mammary” or "mammary Carcinoma*" or "mammary neoplasm*" or "breast carcinoma*" or "mammary tumo*"):ti,ab | 30643 |
| #20 | #18 OR #19 | 31978 |
| #21 | #13 OR #17 | 976899 |
| #22 | #20 AND #21 | 20519 |
| #23 | #4 OR #22 | 21416 |
| #24 | #4 OR #22 with Cochrane Library publication date from Jan 2016 to Dec 2019, in Cochrane Reviews and Cochrane Protocols | 61 |

Database: Epistemonikos

Date: 26.09.2019

Hits: 240

Title/Abstract: ("Magnetic resonance imaging" OR "magnetic resonance imagings" OR "MR scanning" OR "MR scannings" OR "magnetic resonance image" OR "magnetic resonance images" OR "magnetic resonance spectroscopy" OR "magnetic resonance tomography" OR NMR OR NMRs OR MRI OR MRIs OR fMRI OR fMRIs OR "MR tomography" OR "MR Imaging" OR "MR Imagings" OR "MR Image" OR "MR Images" OR "MR scan" OR "MR scans" OR Zeugmatograph* OR "Chemical Shift Imaging" OR "Chemical Shift Imagings" OR "Chemical Shift Image" OR "Chemical Shift Images" OR "Proton Spin Tomography") AND ("Breast Tumour" OR "Breast Tumours" OR "breast malignant tumour" OR "breast malignant tumours" OR "malignant tumour of breast" OR "malignant tumours of breast" OR "mammary tumour" OR "mammary tumours" OR "Breast cancer" OR "Breast Neoplasm" OR "Breast Neoplasms" OR "Breast Tumor" OR "Breast Tumors" OR "breast malignant tumor" OR "breast malignant tumors" OR "malignant tumor of breast" OR "malignant tumors of breast" OR "malignant neoplasm of breast" OR "malignant neoplasms of breast" OR "Mammary Cancer" OR "Cancer of Breast" OR "Cancer breast" OR "breast malignant neoplasm" OR "breast malignant neoplasms" OR "cancer mammary" OR "cancers mammary" OR "mammary Carcinoma" OR "mammary Carcinomas" OR "mammary neoplasm" OR "mammary neoplasms" OR "breast carcinoma" OR "breast carcinomas" OR "mammary tumor" OR "mammary tumors") - limit to: 2016-2019 = 64 (61 Systematic Reviews, 2 Structured Summary, 1 Broad synthesis)

Title/Abstract: ("Magnetization Transfer Contrast Imaging" OR "Magnetization Transfer Contrast Imagings" OR "Magnetization Transfer Contrast Image" OR "Magnetization Transfer Contrast Images" OR "Spin Echo Imaging" OR "Spin Echo Imagings" OR "Spin Echo Image" OR "Spin Echo Images" OR "Diffusion Tractography" OR "Echo Planar Imaging" OR "Echo Planar Imagings" OR "Echo Planar Image" OR "Echo Planar Images" OR "Echoplanar Imaging" OR "Echoplanar Imagings" OR "Echoplanar Image" OR "Echoplanar Images" OR "Magnetic Resonance Angiography" OR “Ultrasound” OR ”Echography” OR "breast imaging" OR "Medical Sonography" OR "Ultrasonic Diagnosis" OR "ultrasonography" OR "ultrasonic imagings" OR Sonography OR "breast imagings" OR "computer echotomagraphy" OR "ultrasonic imaging" OR "sonography medical" OR "Ultrasonic Tomography") AND ("Breast Tumour" OR "Breast Tumours" OR "breast malignant tumour" OR "breast malignant tumours" OR "malignant tumour of breast" OR "malignant tumours of breast" OR "mammary tumour" OR "mammary tumours" OR "Breast cancer" OR "Breast Neoplasm" OR "Breast Neoplasms" OR "Breast Tumor" OR "Breast Tumors" OR "breast malignant tumor" OR "breast malignant tumors" OR "malignant tumor of breast" OR "malignant tumors of breast" OR "malignant neoplasm of breast" OR "malignant neoplasms of breast" OR "Mammary Cancer" OR "Cancer of Breast" OR "Cancer breast" OR "breast malignant neoplasm" OR "breast malignant neoplasms" OR "cancer mammary" OR "cancers mammary" OR "mammary Carcinoma" OR "mammary Carcinomas" OR "mammary neoplasm" OR "mammary neoplasms" OR "breast carcinoma" OR "breast carcinomas" OR "mammary tumor" OR "mammary tumors") - limit to: 2016-2019 = 46 ( 43 Systematic Reviews, 2 Structured Summaries, 1 Broad synthesis)

Title/Abstract: (mammograph* or xeromammograph* or "digital breast tomosynthesis" or "digital breast tomosyntheses") - limit to: 2016-2019 = 130 (121 Systematic Reviews, 4 Structured Summaries, 5 Broad synthesis)

### Search strategy on international guidelines, including overview of relevant hits

Breast cancer screening – search for international guidelines.

Date: 14.02.2020

| Database | Search string(s) | Number of hits | Commentary/ Hit lists |
| --- | --- | --- | --- |
| TRIP+  <http://www.tripdatabase.com/> | **1** mammography  **2** ("Magnetic resonance imaging" OR MRI OR ultrasound) AND "breast cancer" | **1** 159  **2** 440 | **1** [http://www.tripdatabase.com/search?categoryid=16%2C18%2C10%2C9%2C4&criteria=mammography#](http://www.tripdatabase.com/search?categoryid=16%2C18%2C10%2C9%2C4&criteria=mammography)  **2** <http://www.tripdatabase.com/search?categoryid=16%2C18%2C10%2C9%2C4&criteria=(%22Magnetic%20resonance%20imaging%22%20OR%20MRI%20OR%20ultrasound)%20AND%20%22breast%20cancer%22> |
| NHS Evidence in Health and Social Care  <http://www.evidence.nhs.uk/default.aspx> | **1** mammography  **2** ("Magnetic resonance imaging" OR MRI OR ultrasound) AND "breast cancer" | **1** 93  **2** 224 | **1**  [https://www.evidence.nhs.uk/search?om=[{%22ety%22:[%22Guidance%22]}]&q=mammography&sp=on](https://www.evidence.nhs.uk/search?om=%5b%7b%22ety%22:%5b%22Guidance%22%5d%7d%5d&q=mammography&sp=on)  **2** [https://www.evidence.nhs.uk/search?om=[{%22ety%22:[%22Guidance%22]}]&q=(%22Magnetic+resonance+imaging%22+OR+MRI+OR+ultrasound)+AND+%22breast+cancer%22&sp=on](https://www.evidence.nhs.uk/search?om=%5b%7b%22ety%22:%5b%22Guidance%22%5d%7d%5d&q=(%22Magnetic+resonance+imaging%22+OR+MRI+OR+ultrasound)+AND+%22breast+cancer%22&sp=on) |
| G-I-N <https://g-i-n.net/> | **1** mammography  **2** ("Magnetic resonance imaging" OR MRI OR ultrasound OR screening) AND "breast cancer" | **1** 4  **2** 23 | **1**  <https://g-i-n.net/library/international-guidelines-library/@@guideline_search_results?type=basic&basic-searchable-text=mammography>  **2**  <https://g-i-n.net/library/international-guidelines-library/@@guideline_search_results?type=basic&basic-searchable-text=%28%22Magnetic+resonance+imaging%22+OR+MRI+OR+ultrasound+OR+screening%29+AND+%22breast+cancer%22> |
| NICE (UK)  <http://www.nice.org.uk/> | **1** mammography  **2** ("Magnetic resonance imaging" OR MRI OR ultrasound OR screening) AND "breast cancer" | **1** 6  **2** 14 | **1**  [https://www.nice.org.uk/search?om=[{%22ndt%22:[%22Guidance%22]}]&ps=15&q=mammography&sp=on](https://www.nice.org.uk/search?om=%5b%7b%22ndt%22:%5b%22Guidance%22%5d%7d%5d&ps=15&q=mammography&sp=on)  **2**  [https://www.nice.org.uk/search?om=[{%22ndt%22:[%22Guidance%22]}]&ps=15&q=(%22Magnetic+resonance+imaging%22+OR+MRI+OR+ultrasound+OR+screening)+AND+%22breast+cancer%22&sp=on](https://www.nice.org.uk/search?om=%5b%7b%22ndt%22:%5b%22Guidance%22%5d%7d%5d&ps=15&q=(%22Magnetic+resonance+imaging%22+OR+MRI+OR+ultrasound+OR+screening)+AND+%22breast+cancer%22&sp=on) |
| Guideline central  <https://www.guidelinecentral.com/summaries/> | **1** mammography  **2** breast cancer | **1** 1  **2** 36 | **1**  <https://www.guidelinecentral.com/summaries/#term=mammography&type=title>  **2**  <https://www.guidelinecentral.com/summaries/#term=breast+cancer&type=title> |
| UpToDate <https://www.uptodate.com/contents/search> | breast cancer screening | **4**  relevant Up-to-date articles and **1** collection of guidelines from around the world | <https://www.uptodate.com/contents/search?search=breast%20cancer%20screening&sp=0&searchType=PLAIN_TEXT&source=USER_INPUT&searchControl=TOP_PULLDOWN&searchOffset=1&autoComplete=true&language=en&max=10&index=1~10&autoCompleteTerm=Breast%20cancer%20s> |
| WHO IRIS (Institutional repository for information sharing)  <http://apps.who.int/iris> | **1** All of IRIS: mammography  **2** All of IRIS: Magnetic resonance imaging. Filter by Title contains: breast  **3** All of IRIS: MRI. Filter by Title contains: breast  **4** All of IRIS: ultrasound. Filter by Title contains: breast | **1** 503  **2** 3  **3** 5  **4** 11 | **1**  <https://apps.who.int/iris/discover?query=mammography>  **2**  <https://apps.who.int/iris/discover?filtertype_1=title&filter_relational_operator_1=contains&filter_1=breast&submit_apply_filter=&query=magnetic+resonance+imaging&scope=%2F>  **3**  <https://apps.who.int/iris/discover?filtertype_1=title&filter_relational_operator_1=contains&filter_1=breast&submit_apply_filter=&query=MRI>  **4**  <https://apps.who.int/iris/discover?filtertype_1=title&filter_relational_operator_1=contains&filter_1=breast&submit_apply_filter=&query=ultrasound&scope=%2F> |
| European Commission Initiative on Breast Cancer <https://healthcare-quality.jrc.ec.europa.eu/> | **1** mammography  **2** MRI  **3** Magnetic resonance imaging  **4** ultrasound  **5** screening | **1** 50  **2** 7  **3** 7  **4** 9  **5** 118 | The Commission has their own guidelines (<https://healthcare-quality.jrc.ec.europa.eu/european-breast-cancer-guidelines>), as well as a collection on international guidelines on breast cancer care.  **1**  <https://healthcare-quality.jrc.ec.europa.eu/search/node?keys=mammography>  **2**  <https://healthcare-quality.jrc.ec.europa.eu/search/node?keys=MRI>  **3**  <https://healthcare-quality.jrc.ec.europa.eu/search/node?keys=Magnetic+resonance+imaging>  **4**  <https://healthcare-quality.jrc.ec.europa.eu/search/node?keys=ultrasound>  **5**  <https://healthcare-quality.jrc.ec.europa.eu/search/node?keys=screening> |

| **Manual search in Internet (sources with no search engine, or small content)** | | |
| --- | --- | --- |
| **Database** | **Number of relevant hits** | **Commentary** |
| SIGN <http://www.sign.ac.uk/>  (Scotland) | 0 | Looked at the guidelines in the category “Cancer” |
| European Society For Medical Oncology <https://www.esmo.org/guidelines/> | 0 | Looked at the guidelines in the category “Breast Cancer” |
| Ministry of Health - New Zealand  <https://www.health.govt.nz/publications?f%5B0%5D=im_field_publication_type%3A26> | 0 | Looked at the publications in the category “Cancer” |
| CMA INFOBASE  (Canada) <http://www.cma.ca/clinicalresources/practiceguidelines> | 5 | Looked at the guidelines in the category “Breast Cancer”  Complete list is found here: <https://joulecma.ca/cpg/homepage/browse-by/category/conditions/id/68>) |
| CTFPHC (Canadian Task Force on Preventive Health Care) <http://canadiantaskforce.ca/> | 2 | Looked over the guidelines listed. |
| <https://www.cancer.org/healthy/find-cancer-early/cancer-screening-guidelines.html> | 3 | Looked over the guidelines listed. |
| <https://www.cancer.org/health-care-professionals/american-cancer-society-prevention-early-detection-guidelines/breast-cancer-screening-guidelines.html> | 4 | Looked over the guidelines listed. |
| <http://www.who.int/publications/guidelines/en/> | 1 | Looked over the guidelines listed. |
