# Supplementary material for: Conducting a health technology assessment in the West Bank, occupied Palestinian territory: lessons from a feasibility project
Source: Int J Technol Assess Health Care. 2024 Jan 15;40(1):e12. doi: 10.1017/S0266462324000084 (PMC11569958; doi:10.1017/S0266462324000084)
Supplement: Isbeih et al. supplementary material 4 — Isbeih et al. supplementary material [file S0266462324000084sup004.docx]

# Supplementary file 4: Budget Impact Analysis

To produce the Health Technology Assessment (HTA) for Palestine, among other things budget impact analysis (BIA). Below we describe the methods in more detail and provide the search strategy.

### Methods of the Budget Impact Analysis

The objective of the budget impact analysis was to assess the financial implications of implementing mammography screening. We evaluated the screening costs over a five-year period, taking the perspective of the MoH to present the total costs of screening a given population of eligible women per year.

#### The Budget Impact Analysis Model

A deterministic model was created in Microsoft Excel to simulate an organized population-based breast cancer screening program (Figure 1). The model simulates the screening process of an entire population of eligible women in a given year, based on set screening guidelines. The target population included all eligible asymptomatic women according to a given guideline. It was assumed that all women in the target population were offered (or invited) to screen. These women could either participate in the screening program or not (i.e., die from all causes or be lost to follow-up). Women who participated in the program would either get a positive or negative test result. We assumed that women testing positive would be recalled for further testing.

**Figure 1: Deterministic Model**


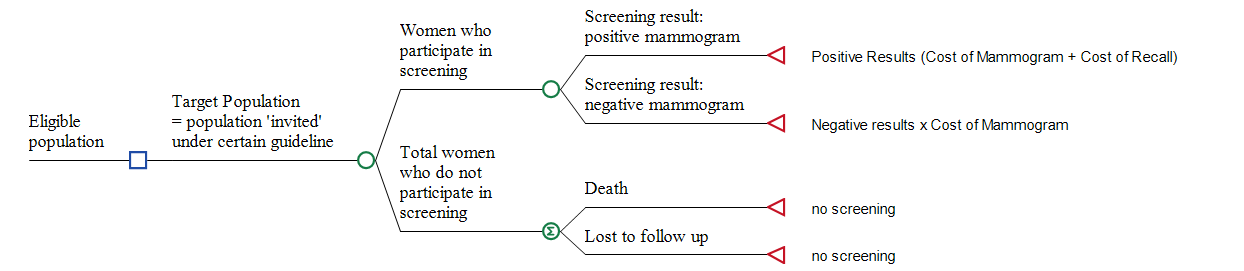


Model was adapted from a similar BIA of breast cancer screening in (1).

The eligible population were asymptomatic women aged 40 and older living in the West Bank. The reason for choosing the youngest age is reflected in the recommendations in the guidelines included in this BIA, where the youngest age included in screening is 40. The eligible population in the first year of the analysis (2019/2020) was estimated to be 307,019 women. An annual growth rate of 2.28% was used to reflect changes in the target population over the five-year period.

We modeled three scenarios based on: 1) the Palestinian screening protocol, 2) the EU protocol and 3) the World Health Organization (WHO) guideline (Table 1). The WHO offers guidelines for limited and well-resourced settings, which were both used in our model.

The Palestinian guidelines recommend screening women aged 40 to 49 years twice a year and 50 to 59 years once a year. The WHO guideline does not make strong recommendations for the younger and older ages groups, however, for this assessment we assumed that most countries would decide to screen once every two years if following the WHO guidelines. For countries with a strong health system the WHO recommends screening all women 40 years and older once every 2 years. For countries with a strong health system but limited resources, the WHO recommends screening once every 2 years. The European Breast Guidelines recommends starting screening at age 45. The recommended frequency is once every 2 years for women 45 to 64 years and once every three years for women older than 70 years.

**Table 1. Population and women covered under a specific guideline**

| **Age interval** | **Population** | | **Guideline interpretations**  *Frequency & Interval women target in screening* | | | |
| --- | --- | --- | --- | --- | --- | --- |
|  | Palestine | West Bank | *WHO well resourced*** | *WHO.limited resources*** | *EU* | *Palestine* |
| **40 - 44** | 114,749 | 75,286 | Once per  2 years | - | - | Once per  *1 year* |
| **45 - 49** | 95,619 | 63,924 | Once per  2 years | - | Once per  2 years | Once per  *1 years* |
| **50 - 54** | 78,084 | 52,416 | Once per  2 years | Once per  2 years | Once per  2 years | Once per  2 years |
| **55 - 59** | 60,053 | 40,129 | Once per  2 years | Once per  2 years | Once per  2 years | - |
| **60 - 64** | 41,847 | 7,360 | Once per  2 years | Once per  2 years | Once per  2 years | - |
| **65 - 69** | 29,928 | 19,528 | Once per  2 years | Once per  2 years | Once per  2 years | - |
| **70 - 74** | 58,290 | 14,985 | Once per  2 years | - | Once per  *3 years* | - |
| **> 75** | 29,145 | 13,391 | Once per  2 years | - | Once per  *3 years* | - |
| **Total*** | 478,570 | 307,019 | 185,483 | *153,510* | *69,717* | *111,137* |

The above numbers include women living in refugee camps, urban, and rural areas. Appendix 1 detailed overview of age groups in five-year intervals.

* The total-row presents the number of eligible women and those women in the target population if an organized screening program was implemented according to a certain guideline.

** WHO well-resourced refers to the recommendations made for well-resourced setting, WHO limited resources refers to the recommendations made for limited resource settings with a strong health system.

Source: Palestinian Central Bureau of Statistics, 2020

We used the average of costs billed for mammography screening and follow-up diagnostic services at health facilities operated by NGOs and the private sector in 2017, as a proxy for average breast cancer screening cost per woman in Palestine. The most recent Consumer Price Index (CPI) for health with the base year 2018 was used to estimate the increase in the proxy-cost (2). The average exchange rate in 2020 was used to convert NIS to US$. We assumed that prices over the five-year period would increase at an inflation-rate of 1.58% (3).

Cost of mammography screening and recall, found in the literature ranged between 80 to 160 NIS (4). In the BIA, we used an average cost of 120.00 NIS (US$ 33.85) per mammogram, and an average cost per recall of 221.11 NIS (US$ 62.36).

**Table 2: Actual Capacity (Diagnostic services used at recall)**

| Technology | Observed capacity | | | | | Price estimates | |
| --- | --- | --- | --- | --- | --- | --- | --- |
|  | Public | NGO | Private | *Total* | *Percentage of total* | Average estimated price (NIS) | **Price per proportion** |
| Breast ultrasounds | 47 | 84 | 88 | *219* | *50,3* | 73,50 | **37,00** |
| Fine needle biopsies | 9 | 36 | 25 | *70* | *16,1* | 250,- | **48,28** |
| MRI* | 10 | 5 | 15 | *30* | *6,9* | 975,- | **67,24** |
| Tru cut biopsy | 15 | 5 | 14 | *34* | *7,8* | 377,50 | **29,51** |
| Histopathology | 7 | 4 | 71 | *82* | *18,9* | 250,- | **47,13** |
| Total |  |  |  |  | 100% |  | **221,11 NIS** |

*No information available for MRI, so rough estimate applied based on theoretical capacity.

Source: Data adapted from Jubran, Shaar, Hammad and Jarrar (2018) (4).

#### Analysis

The parameters used in the model are shown in Table 3. Important parameters for the analysis are the coverage of mammography screening (proportion of eligible women attending), age-specific mortality rates (all-causes), and the recall-rate (i.e., positive mammograms retested or confirmed). Both the estimated attendance and recall rates were assumed to be the same for the whole population and were not expected to change over the five-year horizon. We tested three scenarios of coverage for mammography screening, assuming attendance rates of 20%, 50% and 80%.

To estimate the total cost of screening, the number of women participating in screening was multiplied by the estimated cost per mammogram. The number of women participating in the program was estimated by multiplying the mortality rate and attendance rate with the target population. The model assumed that all women who received a positive result would be re-called for further diagnosis and all attend within the same year. A recall rate of 6.2% was multiplied to estimate the number of women who would be recalled. The total cost of recall was therefore the average cost of diagnostic services during recall multiplied by the number of women recalled.

**Table 3. Input parameters**

| **Parameters** | Estimate | | Source |
| --- | --- | --- | --- |
| **Population Growth rate** | 2.28% | | From [UN](https://population.un.org/wpp/DataQuery/) Department of Economic and Social Affairs, Population dynamics |
| **Coverage of screening** | 80%, 50%, 20% | | Varied coverage estimates in three scenarios​ |
| **Age specific mortality rates** | 40 to 44 | 0.001 | [IHME](http://www.healthdata.org/) on Palestine (2019) |
|  | 45 to 49 | 0.002 |  |
|  | 50 to 54 | 0.004 |  |
|  | 55 to 59 | 0.007 |  |
|  | 60 to 64 | 0.012 |  |
|  | 65 to 69 | 0.022 |  |
|  | 70 to 74 | 0.040 |  |
|  | 75 to 79 | 0.068 |  |
| **Recall rate** | 6.20 % | | Retrospective cohort [study](https://www.pniph.org/public/uploads/Mamography%20Report%20copy.pdf) from Palestine ​(5) |
| **Cost of Mammography** | 120 NIS | | Estimated from  [literature](https://palestine.unfpa.org/en/publications/pathway-survival-story-breast-cancer-palestine) (4)​: *average price charged by NGO and private sector* |
| **Cost of Recall** | 221.11 NIS | | Estimated from [literature](https://palestine.unfpa.org/en/publications/pathway-survival-story-breast-cancer-palestine) (4)​: *average price following on proportion of use diagnostic service* |
| **Consumer Price Index (health)** | 100.55 | | From [Palestinian Central Bureau of Statistics](http://www.pcbs.gov.ps/site/lang__en/695/default.aspx) (3), *average annual consumer price index 2019 (base year: 2018)* |
| **Exchange rate (date)** | 3.565 NIS / US$ | | From the World Bank: *local currency units (Israel) relative to the US Dollar, annual average 2019* |
| **Inflation** | 1.58% | | From WorldData Info (2) |

**References**

1. **Comas M, Arrospide A, Mar J, Sala M, Vilaprinyó E, Hernández C, Et Al.** *Budget Impact Analysis Of Switching To Digital Mammography In A Population-Based Breast Cancer Screening Program: A Discrete Event Simulation Model*. Plos One. 2014;**9**(5):E97459.

2. **Worlddata.Info**. *Development Of Inflation Rates In Palestine*. 2020 [Cited 2021 01-03-2021]; Available From: [Https://Www.Worlddata.Info/Asia/Palestine/Inflation-Rates.Php](https://www.worlddata.info/asia/palestine/inflation-rates.php).

3. **Palestinian Central Bureau Of Statistics**. *Consumer Price Index: Time Series Statistics (Base Year: 2018=100)*. N.D.; Available From: [Http://Www.Pcbs.Gov.Ps/Site/Lang__En/695/Default.Aspx](http://www.pcbs.gov.ps/site/lang__en/695/default.aspx).

4. **Jubran J, Shaar AN, Hammad S, Jarrar K**. *Pathway To Survival - The Story Of Breast Cancer In Palestine*. 2018.

5. **Palestinian National Institute Of Public Health**. *Performance Of Mammography Screening In The National Breast Screening Program: A Retrospective Cohort Study*. World Health Organization,, 2014.
